# Supplementary material for: Volatile profiling in Rhus coriaria fruit (sumac) from three different geographical origins and upon roasting as analyzed via solid-phase microextraction
Source: PeerJ. 2018 Jul 4;6:e5121. doi: 10.7717/peerj.5121 (PMC6035716; doi:10.7717/peerj.5121)
Supplement: Figures S1 and S2 — The S-plot derived from roasted (B) and cold beverage (D) shows the covariance p[1] against the correlation p [1] of the variables of the discriminating component of the OPLS-DA model. Cut-off values of P < 0.05 were used; chosen variables are highlighted in the S-plot with KI values. [file peerj-06-5121-s001.pdf]

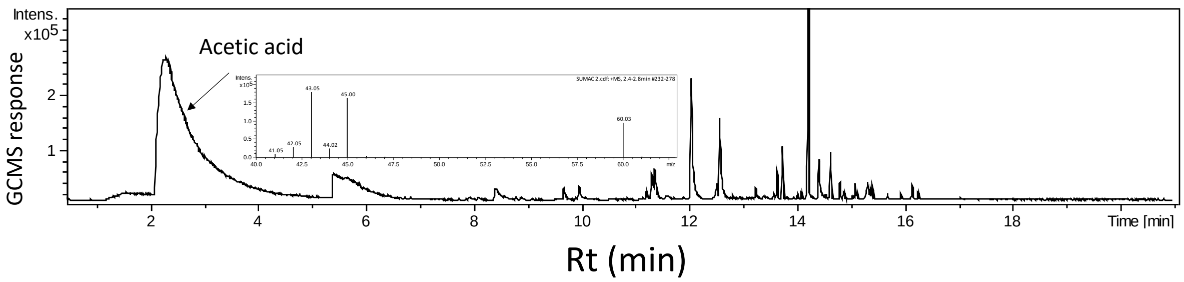

Suppl. Fig. S1: Representative SPME-GC-MS chromatogram of fresh *R. coriaria* fruit (Sumac) starting from 0 minutes and showing major acetic acid peak eluting at 2.3 min.

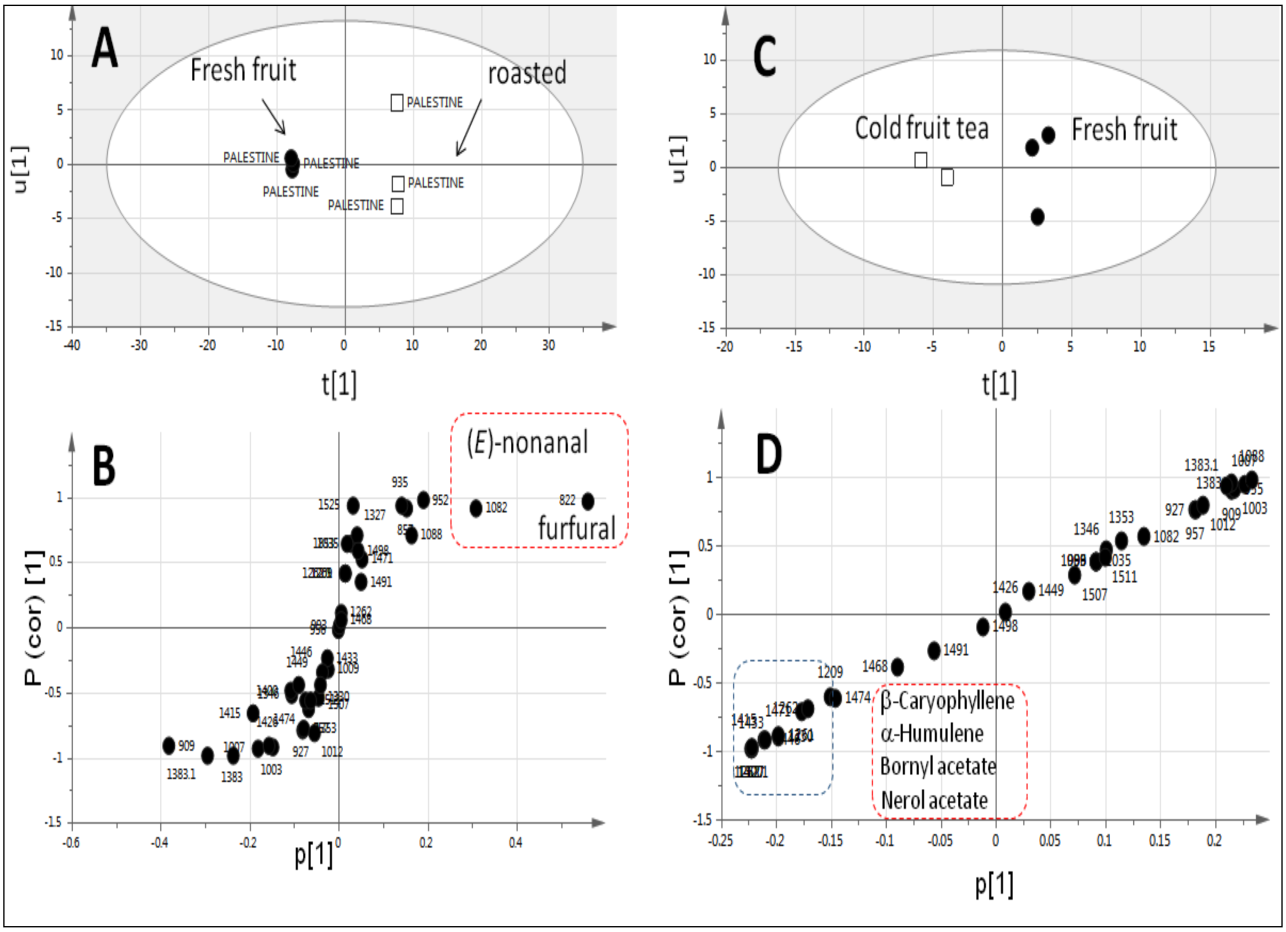

Suppl. Fig. S2: OPLS-DA score plots derived from modeling roasted Sumac fruit (A) and cold beverage Sumac fruit (C) separately against fresh Sumac fruit. The S-plot derived from roasted (B) and cold beverage (D) shows the covariance p[1] against the correlation p [1] of the variables of the discriminating component of the OPLS-DA model. Cut-off values of $P < 0.05$ were used; chosen variables are highlighted in the S-plot with KI values.
